# Overuse of corticosteroids in patients with immune thrombocytopenia (ITP) between 2011 and 2017 in the United States

**DOI:** 10.1002/jha2.684

**Published:** 2023-04-01

**Authors:** Adam Cuker, Joseph Tkacz, Janna Manjelievskaia, Jens Haenig, Joan Maier, James B Bussel

**Affiliations:** ^1^ Department of Medicine and Department of Pathology and Laboratory Medicine Perelman School of Medicine University of Pennsylvania Philadelphia Pennsylvania USA; ^2^ IBM Watson Health Cambridge Massachusetts USA; ^3^ Novartis Pharma AG Basel Switzerland; ^4^ Pediatric Hematology/Oncology Weill Cornell Medicine New York New York USA

**Keywords:** corticosteroids, immune thrombocytopenia, overuse, real‐world evidence, treatment

## Abstract

Corticosteroids (CSs) are standard first‐line therapy for immune thrombocytopenia (ITP). Prolonged exposure is associated with substantial toxicity; thus guidelines recommend avoidance of prolonged CS treatment and early use of second‐line therapies. However, real‐world evidence on ITP treatment patterns remains limited. We aimed to assess real‐world treatment patterns in patients with newly‐diagnosed ITP, using two large US healthcare databases (Explorys and MarketScan) between January 1, 2011 and July 31, 2017. Adults with ITP, ≥12 months of database registration prior to diagnosis, ≥1 ITP treatment, and ≥1 month enrollment following initiation of first ITP treatment were included (*n* = 4066 Explorys; *n* = 7837 MarketScan). Information on lines of treatment (LoTs) was collected. As expected, CSs were the most common first‐line treatment (Explorys, 87.9%; MarketScan, 84.5%). However, CSs remained by far the most common treatment (Explorys ≥77%; MarketScan ≥85%) across all subsequent LoTs. Second‐line treatments such as rituximab (12.0% Explorys; 24.5% MarketScan), thrombopoietin receptor agonists (11.3% Explorys; 15.6% MarketScan), and splenectomy (2.5% Explorys; 8.1% MarketScan) were used much less frequently. CS use is widespread in the US in patients with ITP across all LoTs. Quality improvement initiatives are needed to reduce CS exposure and bolster use of second‐line treatments.

## 1 INTRODUCTION

Immune thrombocytopenia (ITP) is an autoimmune antibody‐mediated hematologic disorder characterized by low platelet counts and increased risk of bleeding, affecting both adults and children [1, 2, 3]. ITP is classified as either primary or secondary to other medical conditions [1, 2, 3]. Pathogenesis involves accelerated antibody‐mediated destruction of platelets and inhibition of megakaryocyte maturation [4]. Spontaneous recovery from primary ITP occurs commonly in children but is less frequent and slower in adults, many of whom progress to chronic disease [5, 6, 7, 8].

First‐line therapy for primary ITP aims to rapidly raise platelet counts and control or prevent bleeding events. Worldwide, the backbone of first‐line therapy is corticosteroids (CSs), supplemented as needed with intravenous immunoglobulin (IVIg), IV anti‐D immunoglobulin, and/or platelet transfusion [1–3, 9]. The majority of patients experience an initial response to CSs, but the response is often transient [3, 10]. Patients who do not respond to first‐line treatment, or who relapse as it is tapered, are candidates for second‐line treatment, including rituximab, thrombopoietin receptor agonists (TPO‐RAs), fostamatinib, and splenectomy as well as other treatments [2, 3, 9].

Treatment guidelines have consistently recommended against prolonged or repeated exposure to CSs because of the substantial side‐effect burden. Indeed, guidelines suggest avoiding steroids and using second‐line therapies in patients with persistent or chronic ITP [2, 3, 11, 12]. This reflects the imperative to avoid overuse of CSs, a recognized issue in ITP management for many years [2, 3, 11, 12]. The 2010 international consensus report highlighted the adverse effects of CS use and advised that prednisone, for example, should be “rapidly tapered and usually stopped even in responders, and especially stopped in nonresponders after 4 weeks.” [12] In 2011, the American Society of Hematology (ASH) guidelines recommended the use of second‐line therapies, including TPO‐RAs and splenectomy, for patients unresponsive to or who relapse after initial CS therapy [11].

This study sought to determine the extent to which such recommendations were followed in real‐world practice by evaluating treatment patterns using data collected for the 2011–2017 period from the IBM Explorys Database and the IBM MarketScan Commercial Claims and Encounters and Medicare Supplemental Databases.

## 2 MATERIALS AND METHODS

A retrospective, observational cohort analysis was conducted to describe treatment patterns in patients with ITP. The data sources for this study were the IBM Explorys Database and the IBM MarketScan Commercial and Medicare Supplemental Databases (full details on study data sources are available in Table S1). Records of adult patients (aged ≥18 years) with at least one inpatient or two outpatient visits carrying a diagnosis of ITP: ICD‐9‐CM 287.31 (International Classification of Diseases, Ninth Revision, Clinical Modification) or ICD‐10 D69.3 (International Classification of Diseases, Tenth Revision) recorded between January 1, 2011 and July 31, 2017, were eligible for inclusion (full details on the study population are provided in Table S1). Eligible patient records were partitioned by treatment type, lines of treatment (LoT), and the sequence of treatments received (full details on study population and selection criteria are available in Table S1). All analyses were descriptive in nature and performed by IBM Watson Health using SAS version 9.4 (SAS Institute) – see Table S1 for full details on statistical methods used. Because Explorys and MarketScan include somewhat different patient populations and data fields, we report results separately for each database rather than pooling results.

## 3 RESULTS

### 3.1 Patient sample

The records of 22,900 and 50,428 patients with ITP during the period January 1, 2011 to July 31, 2017, were identified from the Explorys and MarketScan databases, respectively. Per the inclusion and exclusion criteria detailed in Table 1, the two primary reasons for exclusion were: a) lack of at least 12 months of continuous enrollment prior to the ITP diagnostic index date and b) not having at least one pharmacy or medical claim for ITP treatment following the diagnostic index date. Thus 4066 (17%) patients in Explorys and 7837 (16%) of patients in MarketScan met the eligibility criteria and were included in the analysis (Table 1).

**TABLE 1:** Patient selection.

Patient selection	Explorys	MarketScan
	*n* (%)	*n* (%)
Patients with ≥1 diagnosis code (Explorys) or ≥1 claim^a^ (MarketScan) for ITP between January 1, 2011 and July 31, 2017	22,900 (100)	50,428 (100)
At least 12 months of continuous enrollment prior to the diagnostic index date (baseline period)	11,452 (50.0)	27,119 (53.8)
No evidence of ITP during the 12‐month baseline period	10,539 (46.0)	23,385 (46.4)
At least 1 pharmacy or medical claim for ITP treatment following the diagnostic index date	4834 (21.1)	9268 (18.4)
At least 1 month of continuous enrollment on and following the treatment index date (variable length follow‐up period)	4506 (19.7)	9106 (18.1)
At least 18 years of age on diagnostic index date	4342 (19.0)	8502 (16.9)
No evidence of hepatitis or human immunodeficiency virus, and heparin‐induced thrombocytopenia during the 12‑month baseline period or through the variable‐length follow‐up period	4066 (17.8)	7837 (15.5)

Abbreviations: ITP, immune thrombocytopenia.

^a^ At least one inpatient or two unique outpatient nondiagnostic claims.

Most patients were female (61.0% in Explorys; 59.0% in MarketScan), and the mean ± SD age at start of treatment was 61 ± 18 years and 56 ± 18 years in Explorys and MarketScan, respectively (Table 2). The median (range) time from ITP diagnosis to the start of LoT1 was 45 (0–2298) days in Explorys and 16 (0–2288) days in MarketScan; the mean ± SD length of follow‐up was 1062.1 ± 632.9 days in Explorys and 709.2 ± 568.2 days in MarketScan. Most patients in the Explorys database had either private health insurance (46.4%) or Medicare coverage (35.9%). In the MarketScan database, 68.6% of patients had commercial coverage and 31.4% of patients had Medicare; the most common types of health plan in MarketScan included exclusive provider or preferred provider organization (54.5%) and comprehensive/indemnity insurance (17.0%; Table S2).

**TABLE 2:** Baseline demographics and clinical characteristics.

Patient characteristic	Explorys	MarketScan
	*N* = 4066	*N* = 7837
**Age (years), mean (SD)**	61.1 (18.2)	56.3 (17.7)
**Female, *n* (%)**	2479 (61.0)	4626 (59.0)
**Race, *n* (%)**		
Asian	43 (1.1)	n/a
African American	365 (9.0)	n/a
Caucasian	3108 (76.4)	n/a
Hispanic/Latino	19 (0.5)	n/a
Other	83 (2.0)	n/a
Unknown/Refused	448 (11.0)	n/a
**Treatment index year, *n* (%)**		
Index year 2011–2014	2379 (58.5)	5063 (64.6)
Index year 2015–2017	1687 (41.5)	2774 (35.4)
**Length of follow‐up (days), mean (SD)**	1062.1 (632.9)	709.2 (568.2)
**US geographic region, *n* (%)**		
Northeast	n/a	1524 (19.4)
North central	n/a	2121 (27.1)
South	n/a	3176 (40.5)
West	n/a	966 (12.3)
Unknown	n/a	50 (0.6)
**Deyo‐Charlson Comorbidity Index, mean (SD)**	1.9 (2.5)	2.0 (3.0)

Abbreviations: n/a, not available; SD, standard deviation.

### 3.2 Treatment type across treatment lines

#### 3.2.1 LoT1

**FIGURE 1:**
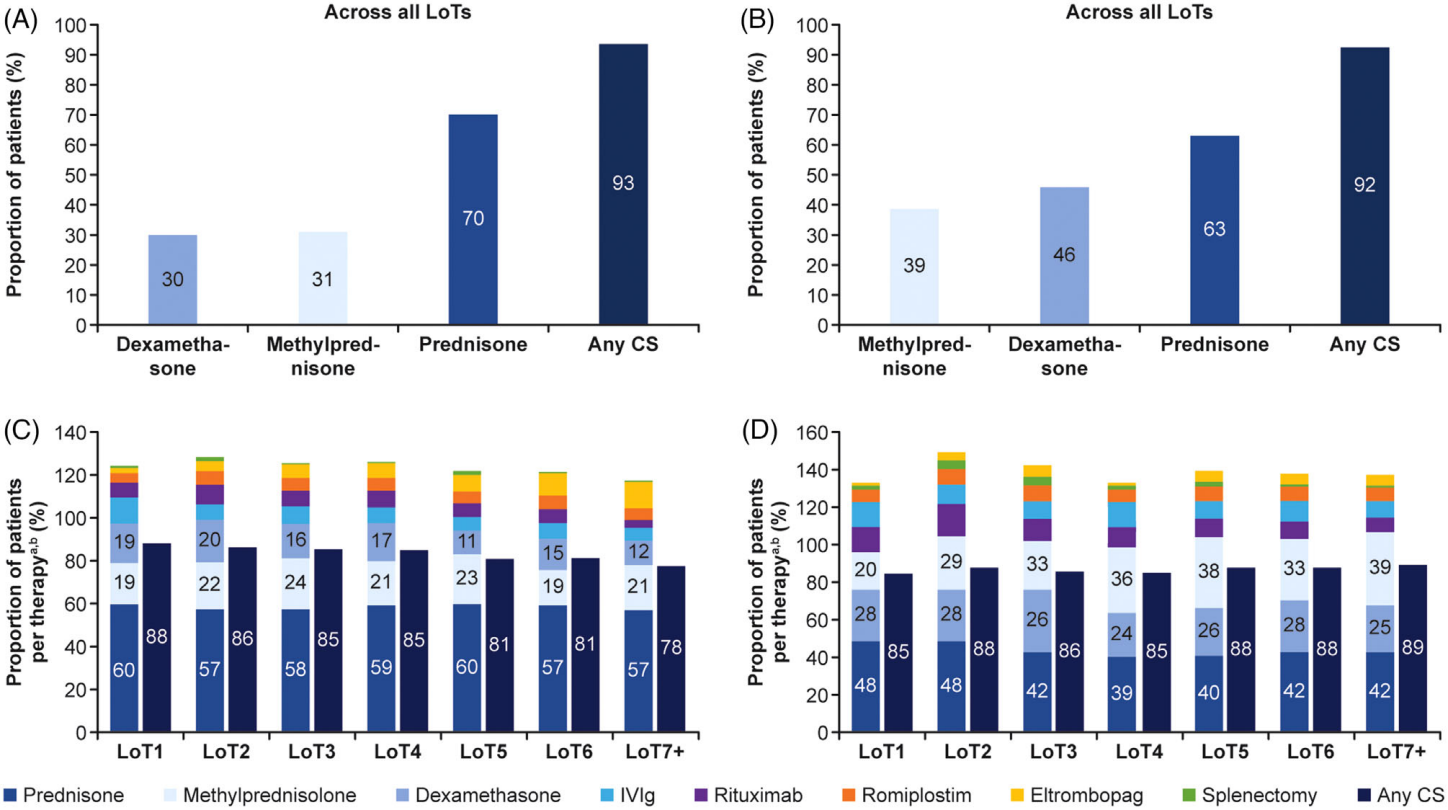
Overall CS use and ITP treatments utilized per LoT in the Explorys and MarketScan databases. Overall CS use across all LoTs in (A) the Explorys database and (B) the MarketScan database. ITP treatments utilized per LoT in (C) the Explorys database and (D) the MarketScan database. ^a^The percentages add to >100% because some patients received more than one treatment in a given LoT. ^b^Treatments presented have been sorted from highest to lowest percentages as used in LoT1 of each database. CS, corticosteroid; ITP, immune thrombocytopenia; LoT, line of treatment.

AS expected, CSs were by far the most common treatment, with most patients receiving CSs in LoT1 (Explorys, 87.9%; MarketScan, 84.5%); more than 90% of patients in both databases received CSs as part of at least one LoT (Figure 1A,B). Of the three CSs included in the analysis, prednisone was the most frequently used in both databases across all patients in any LoT (Explorys, 70.1%; MarketScan, 62.8%); however, methylprednisolone (Explorys, 31.0%; MarketScan, 38.8%) and dexamethasone (Explorys, 29.9%; MarketScan, 45.7%) were also administered frequently. Only 12.3% of Explorys patients and 13.1% of MarketScan patients received IVIg.

In both databases, therapies traditionally reserved for LoT2, such as rituximab and TPO‐RAs, were infrequently administered within LoT1. Rituximab was prescribed to 7.0% and 13.4% of patients, and TPO‐RAs to 6.5% and 8.1% of patients as part of LoT1 in Explorys and MarketScan, respectively. With respect to TPO‐RA use within LoT1, romiplostim was used in 4.4% and 6.6% of patients in Explorys and MarketScan, respectively, and eltrombopag by 2.2% and 1.5% of patients, respectively. Splenectomy was identified in a very small number of patients within LoT 1 in both databases (1.1%, Explorys; 2.5%, MarketScan).

#### 3.2.2 LoT2 and beyond

CS use remained surprisingly high in both databases across LoT2 to LoT7+ ranging from 78% to 86% in Explorys and from 85% to 89% in MarketScan (Figure 1C,D). Repeated courses of CSs were common. In particular, repeated initiation of prednisone (monotherapy or in combination) across the first 3 LoTs (i.e., prednisone → prednisone → prednisone) was by far the most frequent of any treatment sequence among patients receiving at least 3 LoTs (Explorys, 33.0%; MarketScan, 17.8%).

Across all LoTs, second‐line treatments were used much less frequently than CSs (Figure 1C,D). Between LoT2 and LoT7+, rituximab use ranged from 3.5% to 9.1% (Explorys) and from 7.6% to 17.3% (MarketScan) with an overall utilization across all LoTs of 12.0% (Explorys) and 24.5% (MarketScan), and TPO‐RA use ranged from 10.3% to 17.3% (Explorys) and from 10.7% to 13.8% (MarketScan) with an overall utilization across all LoTs of 11.3% (Explorys) and 15.6% (MarketScan). By specific TPO‐RA agent, utilization of eltrombopag ranged from 4.6% to 12.1% (Explorys) and from 4.3% to 7.3% (MarketScan) with an overall utilization across all LoTs of 5.4% (Explorys) and 5.6% (MarketScan). For romiplostim, utilization rates between LoT2 and LoT7+ ranged from 5.3% to 6.4% (Explorys) and from 7.1% to 8.7% (MarketScan), with an overall utilization across all LoTs of 7.3% (Explorys) and 11.8% (MarketScan). Splenectomy use remained low in both databases and ranged from 0.4% to 1.8% (Explorys) and from 1.4% to 4.6% (MarketScan), with an overall utilization across all LoTs of 2.5% (Explorys) and 8.1% (MarketScan). Other immunosuppressive agents (azathioprine, cyclophosphamide, danazol, dapsone, cyclosporine, vinblastine, vincristine, mycophenolate) and hematopoietic stem cell transplant were used rarely across all LoTs.

### 3.3 Treatment effectiveness: Platelet counts in the Explorys database

**FIGURE 2:**
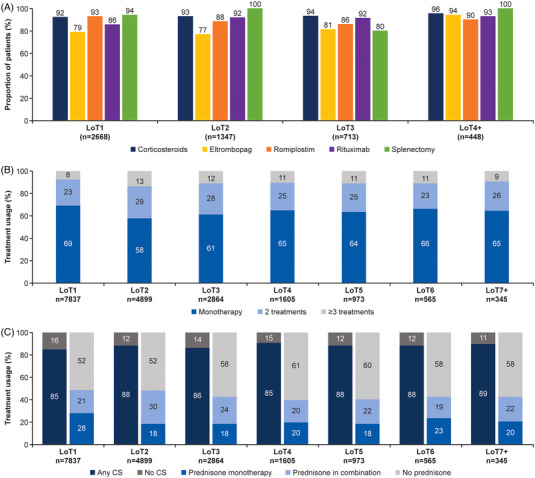
Platelet counts per therapy type and LoT (Explorys database) and treatment regimens per LoT (MarketScan database). The proportion of patients per therapy type and LoT reaching a platelet count of ≥50 ⨯ 10^9^/L in the Explorys database. Patients receiving more than one treatment in a given LoT may be included more than once. (A) Treatment regimens in the MarketScan database, including type of regimen by LoT (B) and prednisone regimen by LoT (C). Regimen type (monotherapy or combination therapy) data were not available for the “Any CS” group. CS, corticosteroid; LoT, line of treatment.

Treatment effectiveness could not be assessed in the MarketScan database because platelet counts were not available. In the Explorys database, 2668 (65.6%) of the 4066 eligible patients were evaluable for platelet response. The great majority of patients in each LoT reached a platelet level of ≥50 ⨯ 10^9^/L (LoT1, *n* = 2446/2668 [91.6%]; LoT2, *n* = 1246/1347 [92.4%]; LoT3, *n* = 660/713 [92.4%]; LoT4+, *n* = 426/448 [94.7%]; Figure 2A). The mean ± SD time from LoT initiation to achieving a platelet count of ≥50 ⨯ 10^9^/L was shortest with LoT1 and longest with LoT4+, with durations of 10.3 ± 30.5 and 13.7 ± 34.2 days, respectively. In contrast, the median time to a platelet count response was 2 days during each LoT, showing that most patients responded rapidly to treatment.

### 3.4 Treatment patterns and steroid use: MarketScan database

For any ITP treatment, in each LoT >50% of patients received monotherapy. Monotherapy treatment regimens were most frequent in LoT1 (*n* = 5424/7837; 69%) and least frequent in LoT2 (*n* = 2835/4899; 58%; Figure 2B).

Because prednisone was the most frequently prescribed treatment, prednisone regimen type was also analyzed. Among LoTs containing prednisone, prednisone was used as monotherapy in 32% (*n* = 1578/4918) and in combination with ≥1 agent in 68% (*n* = 3340/4918). In LoT1, prednisone monotherapy was the most common regimen, with over half of LoT1 prednisone use occurring within a monotherapy regimen (57%; *n* = 2170/3779 patients). Prednisone monotherapy remained a frequently used regimen across all subsequent lines (range, 18%–28%; Figure 2C).

CS use did not appear to be short‐term or low dose. Patients with at least 12 months of continuous enrollment post‐index (*n* = 5170; 66%) were prescribed CSs for a median ± interquartile range of 76 ± 190 days during the first year. Among patients treated with CSs in any given line of treatment (*n* = 5790), only 3.5% of patients were prescribed a prednisone dosage of <5 mg/day (or its equivalent).

### 3.5 Yearly evolution of treatment type: Explorys database

In the Explorys database, treatment patterns over time were examined. Overall, prednisone remained the most widely used treatment in the first, second, and third LoTs throughout the study period. However, between 2015 and 2017, the proportion of patients in Explorys who were treated with eltrombopag, romiplostim, and rituximab as second‐line therapy increased, although patient numbers for these treatments remained low compared with the number of patients treated with CSs (Figure S2).

## 4 DISCUSSION

CSs are an established, cost‐effective first‐line option for treatment of adults with ITP. The initial rate of response to CSs exceeds 70%; however, patients usually relapse upon cessation of treatment and prolonged exposure is associated with toxicity and tolerability issues [2, 3, 9, 12–15]. The well‐documented side effects of long‐term CS use are substantial and wide‐ranging (e.g., mood disturbances, insomnia, weight gain, osteoporosis, opportunistic infections, hypertension); over time, the detrimental effects of CSs far outweigh their benefits [3, 9, 12]. This is reflected in patient experience, with lower overall treatment satisfaction and a greater burden of side effects reported by patients treated with CSs compared with other ITP therapies [16, 17]. Accordingly, both the ASH 2019 guidelines and the International Consensus Report on the Management of ITP recommend against prolonged courses of CSs, with both expert panels urging treatment durations of 6 weeks or less as the preferred LoT1 approach, followed by progression to second‐line options after either lack of response or relapse [2, 3].

In spite of the ASH 2011 guidelines [11] and 2010 consensus report recommendations [12] to substantially limit duration of steroid use, the results reported here show that CS overuse for treatment of patients with ITP was rampant in clinical practice in the United States from 2011 to 2017, not only as expected in LoT1, but across all subsequent LoTs through LoT7+. This pattern of overuse was surprisingly consistent in both databases through all LoTs examined and was maintained from 2011 through to 2017. Data from the MarketScan databases indicate that high dosages of CSs were often used (few patients [3.5%] received <5 mg/day prednisone or its equivalent) and the median duration of CS treatment, as assessed by prescription, was 76 days (approximately 11 weeks) during the first year, considerably longer than the ≤6 weeks of steroid use currently recommended in the ASH guidelines [2].

Our data expand on the findings of other studies that have evaluated CS use in patients with ITP. For example, the TIMES study of Spanish registry data from 2009 to 2011 reported that the duration of CS treatment exceeded 6 weeks in the majority of patients with primary ITP [18]. Real‐world patient data from a Korean Health Insurance Database between 2010 and 2014 showed that CSs were not only the most frequently used therapy after 3 months of treatment in patients with ITP but were also used in more than half of patients with ≥48 months of follow‐up [19].

Why are CSs so consistently overused contrary to multiple sources of guidance? Perhaps because they are effective, inexpensive, easy to administer, and appear tolerable, at least in the short‐term [2, 10]. It is also important to acknowledge the role of medical insurance in shaping treatment decisions. Insurance approvals for second‐line agents and appointments at infusion centers can take days or weeks to obtain, meaning that if a patient relapses after tapering CSs (and the physician does not wish to hospitalize the patient), the simplest and fastest solution may be to resume or increase the dose of CSs. Additionally, the perception that second‐line therapies may be effective but are not curative coupled with the cost of treatment may have contributed to driving treatment selection in favor of CSs.

Analysis of treatment utilization by year in the Explorys database indicated that use of CS remained consistently high from 2011 to 2017, with only a small decrease in CS use from LoT2 in 2017. The use of second‐line therapies such as TPO‐RAs showed a slight increase in use from LoT2 onward in 2017. This may be because limiting CS use was not emphasized nearly as strongly in the 2010 international consensus report and ASH 2011 guidelines (which were in place during the study timeframe) as in the current 2019 versions [11, 12]. Furthermore, although eltrombopag and romiplostim were both approved in 2008, the US Food and Drug Administration instituted a risk evaluation and mitigation strategy for both drugs with consent requirements, restrictions regarding distribution, and mandatory safety data collection. This likely slowed the uptake of TPO‐RA agents, at least during the first part of the study period [20, 21, 22, 23].

While the results demonstrate overall prolonged CS use, some patients may have received only brief courses of CSs to limit side effects (such as infusion reactions) or to rapidly raise platelet counts while waiting for other treatment(s) to take effect. Given that only treatment prescription rather than treatment use was assessed, patients may not have used the full complement of prescribed medication (with CSs possibly being over‐prescribed as a precautionary “backup” measure), potentially inflating CS and combination therapy usage rates in this study [3, 11].

Current treatment guidelines for ITP recommend second‐line options such as TPO‐RAs, rituximab, and splenectomy over CSs for the treatment of ITP beyond first line [2, 3]. Fostamatinib, although licensed too late to be included in the ASH guidelines, is another second‑line option. The effectiveness of second‐line agents is reflected in the current study: in the Explorys database for each treatment type (CSs, TPO‐RAs, rituximab, or splenectomy), >77% of patients achieved a platelet level of ≥50×10^9^/L in each LoT. Nonetheless, second‐line therapies were not used nearly as frequently as one might expect, given clinical guidelines, international consensus recommendations, and ample published academic discourse on the management of patients with ITP.

Of note, splenectomy was performed in LoT1 in 1%–2% of patients (Figure S1D) suggesting that 1%–2% may be the approximate incidence of so‐called refractory ITP at presentation. This is aligned with the ASH guidelines, which suggest delaying splenectomy for at least 1 year after diagnosis, if possible [2].

The databases chosen for this study were selected because they provide a large sample of the US population with high longitudinal integrity, capturing a detailed overview of patient encounters from pre‐hospitalization through any inpatient care to any post‐hospitalization visits or outpatient pharmaceutical claims. Additionally, the data are based on claims that have been fully paid or adjudicated, which increases their accuracy. However, these databases have important limitations, including the requirement for continuous enrollment in an insurance plan, limited demographic information, and difficulty in linking treatment to platelet counts [24]. In addition to the limitations of the Explorys and MarketScan databases, there are also limitations inherent with any medical claims data analysis. First, the medication usage results are based on administrative claims and reflect prescription of patient medication, not actual patient use. Second, different databases often capture different underlying data thereby limiting the ability to perform the same analyses across multiple databases to corroborate results. Third, there is variability in the diagnostic decision criteria used by providers and the amount of patient‐reported information during each visit. Also, the costs of and access to treatments may vary, and results reported here may not be generalizable to patients with ITP who have different types of health coverage. The data in the current study reflect the years 2011–2017, a time prior to the current 2019 ASH guidelines and 2019 international consensus report. These data were also collected before the COVID‐19 pandemic. It is not clear how the pandemic has affected CS use in patients with ITP.

Another limitation of the current study is that the numbers of patients captured in the records, especially after LoT2, decreased dramatically. This may be because patients recovered and no longer needed treatment (an outcome that occurs in at least 30% of adult patients with ITP) [25, 26]. However, this may also reflect the study inclusion criteria, which only required eligible patients to have 1 month of follow‐up after the treatment index date, thereby skewing the data toward shorter follow‐up durations. Other possibilities include changing to a physician outside the reporting network or a patient deciding not to monitor their platelet count or not to receive treatment despite a low platelet count.

Finally, it is worth noting that the vagaries of the US health insurance system may have played a role in the overuse of steroids. Interestingly, the treatment patterns in the Explorys database, which includes Medicaid and Medicare, revealed less use of each of the second‐line therapy options: eltrombopag, romiplostim, rituximab, and even splenectomy compared with the treatment patterns observed based on the MarketScan data.

In summary, this study documents substantial overuse of CSs in adults with ITP from 2011 to 2017. Similar patterns of CS overuse were demonstrated in both databases and, most compellingly, across every line of treatment. Quality improvement efforts are needed to curtail overuse of CSs, bolster earlier use of CS‐sparing second‐line therapies, and bring current clinical practice into alignment with the ASH guidelines and the ICR [2, 3, 18, 19, 27, 28].

## AUTHOR CONTRIBUTIONS

**Table:** 

	AC	JT	J Manjelievskaia	JH	J Maier	JBB
**Conceptualization/Study design**	X	X	X	X	X	X
**Data collection**		X	X			
**Data analysis**			X			
**Interpretation of results**	X	X	X	X	X	X
**Manuscript writing, reviewing, and editing**	X	X	X	X	X	X
**Other (please specify)**						

## CONFLICT OF INTEREST STATEMENT

A. Cuker served as a consultant for Synergy and has received authorship royalties from UpToDate. J. Tkacz received research funding from Novartis to conduct this study. J. Manjelievskaia received research funding from Novartis to conduct this study. J. Haenig employed by Novartis Pharma AG. J. Maier employed by Novartis Pharma AG, owner of Novartis shares. J.B. Bussel is consultant to Amgen, Argenx, AstraZeneca, Janssen Pharmaceuticals, Novartis, Rigel Pharmaceuticals, RallyBio, Sobi, UCB and is Member of a data safety and monitoring board for UCB.

## ETHICS STATEMENT

The study uses only de‐identified patient records fully compliant with United States patient confidentiality requirements and does not involve the collection, use, or transmittal of individually identifiable data.

## Supporting information

Supporting Information

Supporting Information

Supporting Information

## Data Availability

Novartis is committed to sharing with qualified external researchers’ access to summary‐level data. These requests are reviewed and approved by an independent review panel on the basis of scientific merit. All data provided are from IBM Watson Health.
