# Supplementary figures and images for: Overuse of corticosteroids in patients with immune thrombocytopenia (ITP) between 2011 and 2017 in the United States
Source: EJHaem. 2023 Apr 1;4(2):350–7. doi: 10.1002/jha2.684 (PMC10188501; doi:10.1002/jha2.684)

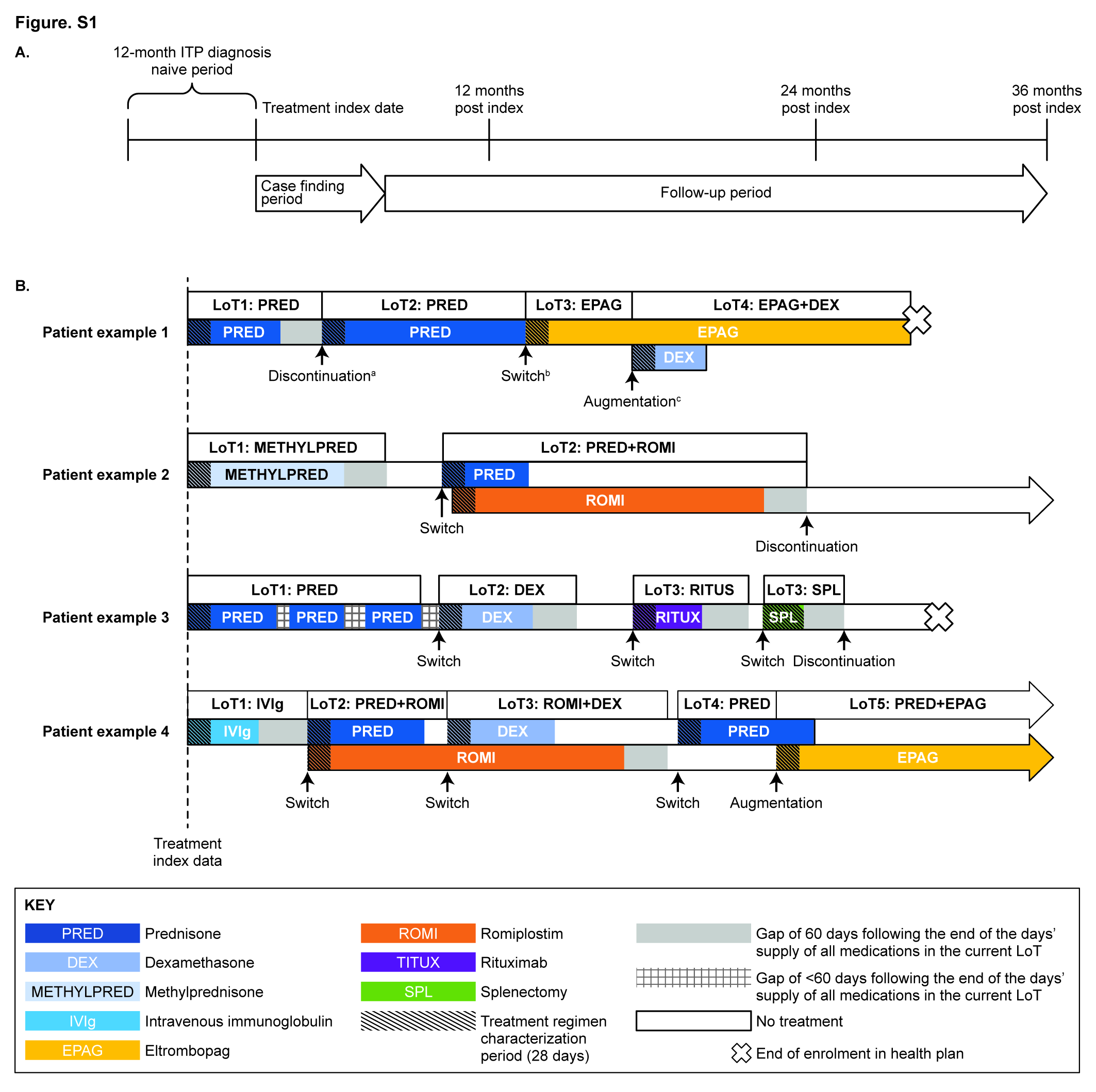

Supplement: Supplementary file 1 — Supporting Information [file JHA2-4-350-s001.tif]

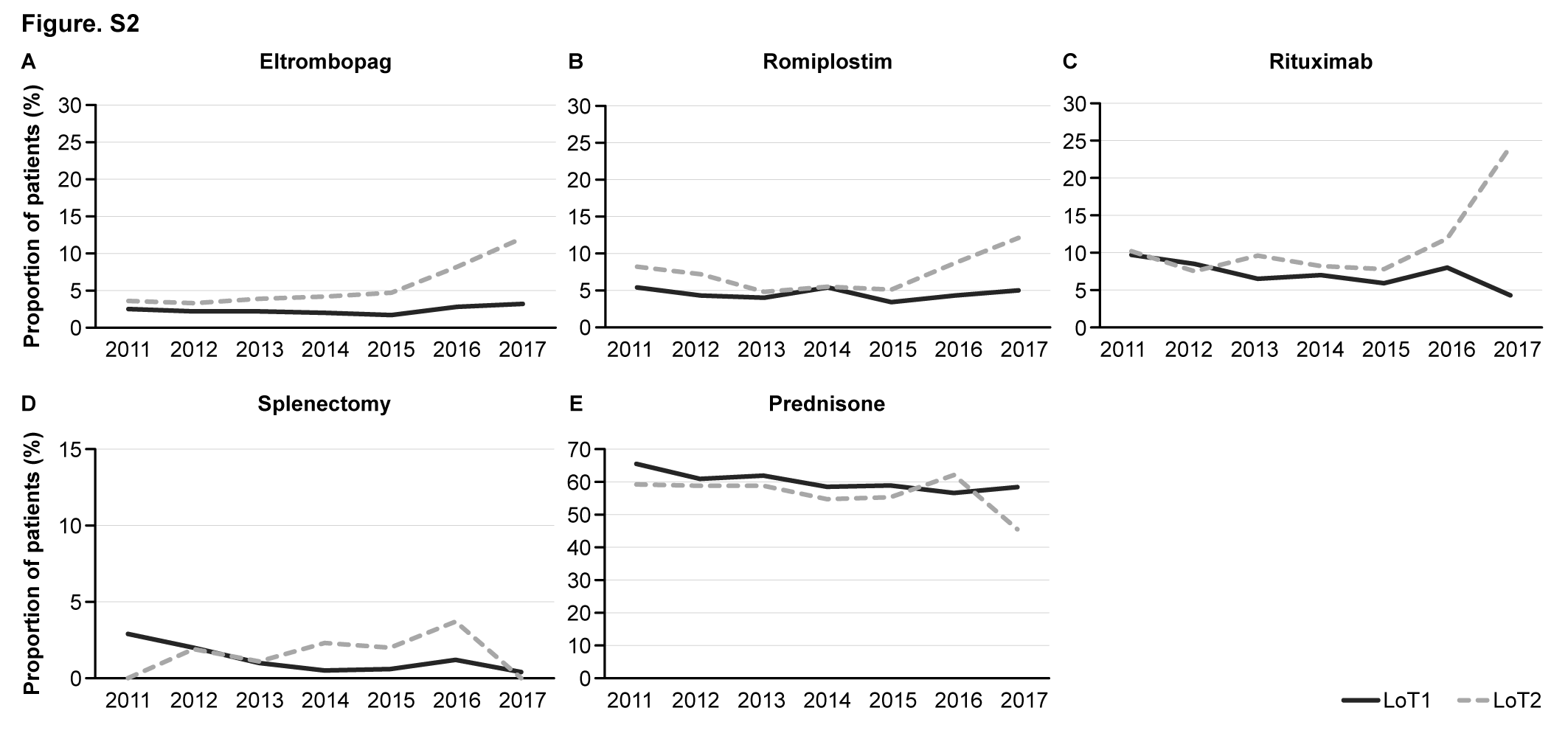

Supplement: Supplementary file 2 — Supporting Information [file JHA2-4-350-s002.tif]
