# Supplementary material for: Overuse of corticosteroids in patients with immune thrombocytopenia (ITP) between 2011 and 2017 in the United States
Source: EJHaem. 2023 Apr 1;4(2):350–7. doi: 10.1002/jha2.684 (PMC10188501; doi:10.1002/jha2.684)
Supplement: Supplementary file 3 — Supporting Information [file JHA2-4-350-s003.docx]

## Supplemental figure legends

## Figure S1. Study design and theoretical examples of patients’ lines of therapy

(A) Overview of the overall study design; (B) Theoretical examples illustrating patients’ lines of therapy. Treatment durations are not shown to scale. ^a^Discontinuation is defined as a gap of ≥60 days following the run-off date of the last observed claim. ^b^Switching is defined as the addition of a new ITP treatment after discontinuing (gap of ≥60 days) all agents of the most recent line of therapy or, in the case of some combination therapy regimens, the start of a new treatment following the discontinuation of a treatment that was present in the previous line). ^c^Augmentation is defined as the addition of a new therapy ≥28 days after initiation of a treatment regimen.

DEX, dexamethasone; EPAG, eltrombopag; ITP, immune thrombocytopenia; IVIg, intravenous immunoglobulin; LoT1, first line of treatment; METHYLPRED, methylprednisone; PRED, prednisone; RITUX, rituximab; ROMI, romiplostim; SPL, splenectomy

## Figure S2. Use of second-line treatments in LoT1 and LoT2 by year (Explorys database)

LoT, line of treatment; N, total number of patients across all treatments.

| **Supplemental tables**  **Table S1: Study methodology (data sources, study population, study design, study outcomes, statistical analysis)** | |
| --- | --- |
| **Data sources** | The data sources for this study were the IBM® Explorys Database and the IBM® MarketScan® Commercial and Medicare Supplemental Databases. The Explorys database consists of data on approximately 55 million patients in the United States (US). It contains data from electronic health records, adjudicated claims, and outgoing billing. The MarketScan databases contain fully adjudicated medical and pharmacy claims for approximately 50 million patients living in the US. All Explorys and MarketScan database records are de-identified and fully compliant with US patient confidentiality requirements.  The Explorys database provides healthcare data for US patients insured commercially, or as part of the national Medicaid or Medicare programs. The MarketScan database contains the inpatient, outpatient, and outpatient prescription-drug experience of US employees and their dependents, covered under a variety of fee-for-service and capitated health plans. The Explorys database was used for assessing treatment effectiveness outcomes because of the inclusion of electronic health records; treatment utilization patterns were assessed in both databases. Additionally, analyses of the Explorys and MarketScan data differed because of the underlying differences in the data captured by each database. The MarketScan database was used to analyze overall treatment regimen type (e.g. monotherapy vs combination therapy), corticosteroid dose and duration, and reasons for LoT changes (e.g. rates of switching, augmentation, and discontinuation). The Explorys database was used for platelet count data and treatment utilization patterns over time. The analyses of the two databases were conducted separately. Because of the study design and the evolving nature of the database populations, the possibility exists that a very small fraction of patients may have been included in both databases. |
| **Study population** | Records of adult patients (aged ≥18 years) with at least one inpatient or two outpatient visits carrying a diagnosis of ITP: ICD-9-CM 287.31 (International Classification of Diseases, Ninth Revision, Clinical Modification) or ICD-10 D69.3 (International Classification of Diseases, Tenth Revision) recorded between January 1, 2011 and July 31, 2017, were eligible for inclusion. The first encounter including an ITP diagnosis was considered the diagnostic index date. To ensure that patients were newly-diagnosed patients, they were required to have at least 12 months’ enrollment without an ITP diagnosis prior to the diagnostic index date. The date of the earliest evidence of ITP treatment was considered the treatment index date. Patient records were required to contain evidence of at least one ITP treatment after diagnosis and at least 1 month of available data after the treatment index date. Patients with hepatitis, human immunodeficiency virus, and/or heparin-induced thrombocytopenia diagnoses were excluded. |
| **Study design** | A retrospective, observational cohort analysis was conducted. The study period extended from January 1, 2011 to July 31, 2017 and included patient selection and outcome assessment. The time period prior to the initial diagnosis of ITP, along with a variable-length follow-up period for the assessment of study endpoints, was included (Figure S1A).  Eligible patient records were partitioned by treatment type, LoT, and the sequence of treatments received. Qualifying ITP treatments included prednisone, dexamethasone, methylprednisolone, IVIg, IV anti-D immunoglobulin, rituximab, eltrombopag, romiplostim, azathioprine, cyclophosphamide, danazol, dapsone, cyclosporine, vinblastine, vincristine, mycophenolate, splenectomy, and hematopoietic stem cell transplant. Avatrombopag and fostamatinib were not included because they were not approved until after the end of the study period.13,14 Patients were assumed to have completed the prescribed supply of medication.  LoTs were identified, and treatment sequences were constructed, starting with the index treatment regimen through the end of the variable length follow-up period. Fictional examples of patients’ treatment pathways are shown as illustrations in Figure S1B.  LoT1 started on the date of the first ITP treatment (treatment index date). Treatments received within the first 28 days from the start of LoT1 were used to characterize the treatment regimen including changes to the treatment regimen during this time. Changes to the treatment regimen occurring after the initial 28-day characterization period triggered the end of the current LoT and, if treatment was continued but changed, triggered the start of the subsequent LoT, e.g. LoT2.  A list of potential changes to the treatment regimen, which would thereby trigger the end of a LoT, are defined below:  a) Augmentation: the addition of a new therapy ≥28 days after initiation of a LoT (if a second medication is started within the first 28 days of treatment, the LOT is not changed). For example, a patient on treatment X who started additional treatment Y > 28 days after treatment X started, would trigger a new LoT. The subsequent stopping of either treatment X or treatment Y, would not trigger another LoT change (see discontinuation below).  b) Discontinuation: a gap of ≥60 days following the completion of the prescribed number of days of supply of all medications in the current LoT. Discontinuation does not occur if a patient discontinues temporarily (<60 days): if a patient discontinued treatment X only to resume the same treatment a month later, the entire period of treatment with medication X were considered part of the same LoT. If an LoT consisted of 2 treatments e.g. X and Y, both would need to be stopped for > 60 days to trigger a new LoT (if treatment of any kind was subsequently administered).  c) Switching: the initiation of a new ITP treatment after discontinuing all other treatments if the gap between treatments was ≥60 days. If the gap was >28 days but <60 days, a new LoT was started per the definition of augmentation and discontinuation above. For example, a patient discontinued treatment X and commenced treatment Y, or the start of a new treatment following the discontinuation of a treatment agent that was present in the previous line, e.g., a patient who received a combination of treatment X and treatment Y, discontinued treatment Y in favor of treatment Z. The new treatment regimen, switched from “XY” to “XZ”, is a new LOT.  d) Health plan disenrollment.  e) Patient death; or,  f) End of study (July 31, 2017)  Each successive LoT began with a new 28-day characterization period that started on the date of commencing a new treatment that was not part of the previous line (in the event of a switch or an augmentation) or on the restart date of treatment that was previously discontinued for >60 days. Because of the lower number of patients in later LoTs, patients that received treatment in LoT7 and beyond were grouped for analysis. |
| **Study outcomes** | As specified in the protocol, the objective of the study was to assess the effectiveness of specific ITP treatments. However, because limited platelet count was data available, the primary focus shifted to a descriptive analysis of the treatment patterns experienced by patients in the Explorys and MarketScan databases. Monotherapy and combination therapy regimens could not be analyzed because treatment regimen information was not available in the Explorys databases. Only filled prescriptions were included. Within both databases, the utilization of each treatment agent and the most frequent treatment sequences were analyzed. |
| **Statistical analysis** | All analyses were descriptive in nature and performed by IBM Watson Health using SAS version 9.4 (SAS Institute). Descriptive statistics were calculated separately for the Explorys and MarketScan databases including means, medians, and standard deviations (SDs); minimum and maximum values for continuous variables; and frequencies and percentages for categorical variables. The percentages for total number of patients across all medications could be >100% because some patients received more than one treatment in a given LoT. Treatment type by LoT analysis was conducted to identify the most frequently used treatment sequences. As such, any patients receiving multiple treatments in a given LoT would be included in the counts for each treatment. |

## Table S2. Health plan types included in the Explorys and MarketScan^®^ databases

| **Explorys database** |  |
| --- | --- |
| **Health plan type, *n* (%)** | ***N* = 4066** |
| Private | 1885 (46.4) |
| Medicare | 1459 (35.9) |
| Medicaid | 189 (4.6) |
| Self-pay | 202 (5.0) |
| Other/unknown | 331 (8.1) |

| **MarketScan database** |  |
| --- | --- |
| **Primary payer, *n* (%)** | ***N* = 7837** |
| Commercial | 5380 (68.6) |
| Medicare | 2457 (31.4) |
| **Health plan type, *n* (%)** | ***N* = 7837** |
| Comprehensive/indemnity | 1336 (17.0) |
| Exclusive provider organization/Preferred provider organization | 4268 (54.5) |
| Point-of-service/Point-of-service with capitation | 533 (6.8) |
| Health maintenance organization | 733 (9.4) |
| Consumer-directed health plan/High deductible health plan | 757 (9.7) |
| Missing/unknown | 210 (2.7) |
